# Supplementary material for: Identification and Expression Analysis of SLAC/SLAH Gene Family in Brassica napus L
Source: Int J Mol Sci. 2021 Apr 28;22(9):4671. doi: 10.3390/ijms22094671 (PMC8125795; doi:10.3390/ijms22094671)
Supplement: Supplementary file 1 [file ijms-22-04671-s001.zip › ijms-1151624-supplementary.pdf]

Identification and Expression Analysis of *SLAC/SLAH* Gene Family in *Brassica napus* L.

Supplementary materials

Table S1. *SLAC/SLAH* genes in the *Brassica rapa* genome

Gene ID	Gene name	Chromosome	Start location (bp)	Stop location (bp)
LOC103865761	*BrSLAH3-2*	A09	3670394	3675192
LOC103874270	*BrSLAH3-1*	A06	21648699	21654702
LOC103854877	*BrSLAH3-3*	A02	29417653	29422408
LOC103834780	*BrSLAH2-2*	A08	15000333	15003041
LOC103867762	*BrSLAH2-3*	A01	10012171	10019301
LOC103834360	*BrSLAH2-1*	A03	28016210	28018952
LOC103871999	*BrSLAC1-1*	A06	4998188	5000423
LOC103848156	*BrSLAH1-1*	A03	36546634	36556724
LOC103838028	*BrSLAH1-2*	A09	7332242	7334989
LOC103838410	*BrSLAH1-3*	A09	9759409	9772631
LOC103838413	*BrSLAH4-1*	A09	9775220	9789528

All *SLAC/SLAH* genes in *Brassica rapa* are named as *BrSLAC/SLAH* according to the order of closest orthologues in *Arabidopsis.*

Table S2. *SLAC/SLAH* genes in the *Brassica oleracea* genome

Gene ID	Gene name	Chromosome	Start location (bp)	Stop location (bp)
LOC106318874	*BoSLAH3-2*	C09	4008913	4013581
LOC106301292	*BoSLAH3-1*	C07	37799902	37804661
LOC106310325	*BoSLAH2-2*	C08	17709299	17711960
LOC106304635	*BoSLAH2-1*	C07	44486030	44488377
LOC106326919	*BoSLAH2-3*	C01	14469888	14472390
LOC106295057	*BoSLAC1-1*	C05	5418536	5420688
LOC106340717	*BoSLAH1-1*	C04	22530746	22532251
LOC106316982	*BoSLAH4-1*	C09	13136998	13143186
LOC106340224	*BoSLAH4-2*	C04	26970788	26972968
LOC106317353	*BoSLAH1-2*	C09	13158561	13159882
LOC106322198	*BoSLAH4-3*	Unplaced		
LOC106338400	*BoSLAH4-4*	C04	22423431	22424981

All *SLAC/SLAH* genes in *Brassica oleracea* are named as *BoSLAC/SLAH* according to the order of closest orthologues in *Arabidopsis*.

Table S3. Primers used for gene cloning and qRT-PCR

Primer name	Sequences(5'-3')	Functions
BnSLAH1-1-FP	ATGGAAAGCTTGGAAATTCCT	Gene cloning
BnSLAH1-1-RP	ACATGTACCAGTGCCGAGCCG	Gene cloning
BnSLAH3-2-FP	ATGGAGGGGAGATCAAATCGT	Gene cloning
BnSLAH3-2-RP	AGCCGAATCCTTCTCTTGAAT	Gene cloning
BnSLAH3-3-FP	ATGGAGGAGAGATCAAGTTGT	Gene cloning
BnSLAH3-3-RP	TGCGGAATCACTCTCTTGAGC	Gene cloning
q*Bnactin7*-FP	TGTTTGGATTGGAGGATCCATCTTG	qRT-PCR
q*Bnactin7*-RP	GTGGACGATGGATGGACCCG	qRT-PCR
q*BnSLAC1-1*-FP	TGGCGTGGTGGTCATATACT	qRT-PCR
q*BnSLAC1-1*-RP	GCCGAGGAAATGAAGGAGAGAG	qRT-PCR
q*BnSLAH1-1*-FP	ACGGGGAGCAGCAGAGATA	qRT-PCR
q*BnSLAH1-1*-RP	TACCTCCCGGTAGACGTTGA	qRT-PCR
q*BnSLAH3-2*-FP	TGCAAGCATGGGACTTAGGG	qRT-PCR
q*BnSLAH3-2*-RP	GTCTCGTTGGTCGGTAACCT	qRT-PCR
q*BnSLAH3-3*-FP	GTCCCACCTTCCATAGCTACA	qRT-PCR
q*BnSLAH3-3*-RP	ACCACCGGACATCCATTGAC	qRT-PCR
q*BnSLAH3-4*-FP	CGTACGCCGAGAATACTACCA	qRT-PCR
q*BnSLAH3-4*-RP	AGGAACTGTGGCAACTCCTTAG	qRT-PCR

Table S4. The FPKM values of expression levels of *BnSLAC/SLAH* genes in different tissues.

Gene name	root	stem	leaf	flower	sepal	pistil	blossomy pistil	wilting pistil	stamen	ovule	silique	pericarp
BnSLAH2-1	0	0	0	0	0	0	0	0	0	0	0	0
BnSLAH2-4	0.00	0.00	0.00	0.00	0.00	0.00	0.00	0.00	0.00	0.00	0.00	0.00
BnSLAH3-1	0.00	0.00	0.00	0.00	0.00	0.00	0.00	0.00	0.00	0.00	0.00	0.00
BnSLAH2-5	0.00	0.00	0.00	0.00	0.00	0.00	0.00	0.00	0.00	0.00	0.00	0.00
BnSLAH4-6	0.00	0.00	0.00	0.00	0.00	0.00	0.00	0.00	0.00	0.00	0.00	0.00
BnSLAH4-5	0.00	0.06	0.00	0.00	0.00	0.00	0.00	0.00	0.00	0.00	0.00	0.00
BnSLAH3-6	0.05	0.00	0.02	0.36	0.00	0.00	0.00	0.00	0.08	0.97	0.03	0.33
BnSLAH2-3	0.10	0.00	0.00	0.03	0.00	0.00	0.00	0.00	0.00	0.00	0.00	0.00
BnSLAH4-4	0.10	0.00	0.09	0.00	0.00	0.00	0.00	0.28	0.00	0.00	0.00	0.00
BnSLAH1-2	0.00	0.00	0.00	7.64	0.00	26.01	0.00	0.00	34.05	0.00	0.46	1.77
BnSLAC1-2	0.93	1.15	2.88	1.36	9.62	0.48	0.63	0.68	0.46	0.73	1.22	2.39
BnSLAH3-2	9.30	4.49	1.03	2.51	7.98	7.83	99.87	22.74	8.41	10.28	6.08	4.97
BnSLAH4-3	0.04	0.00	0.00	0.10	0.00	0.00	0.00	0.00	0.00	0.06	0.00	0.00
BnSLAH3-3	16.65	3.70	10.91	2.36	7.99	6.68	18.53	18.36	12.51	0.39	9.11	8.46
BnSLAH4-2	0.07	0.00	0.00	0.07	0.00	0.00	0.00	0.00	0.00	0.00	0.00	0.00
BnSLAH1-4	0.00	0.00	0.00	0.04	0.00	0.00	0.00	0.00	0.06	0.00	0.17	0.00
BnSLAH3-4	34.84	6.02	8.44	0.52	5.80	1.32	85.43	41.18	18.74	0.22	3.04	8.56

BnSLAH3-5	0.03	0.00	0.05	3.36	0.15	0.09	0.00	0.05	2.18	3.36	0.43	0.00
BnSLAC1-1	2.45	1.53	3.38	1.83	6.23	1.30	1.00	0.94	0.62	0.82	1.66	2.74
BnSLAH2-2	0.48	2.12	0.03	0.00	0.05	0.05	0.00	0.00	0.00	0.00	0.22	0.17
BnSLAH1-1	0.21	0.00	0.00	1.07	0.32	7.52	3.24	1.26	266.69	0.42	0.00	0.00
BnSLAH1-3	0.04	0.00	0.00	0.00	0.00	0.00	0.00	0.00	0.51	0.00	0.00	0.00
BnSLAH4-1	0.03	0.00	0.00	0.00	0.00	0.12	0.00	0.00	0.00	0.00	0.00	0.00

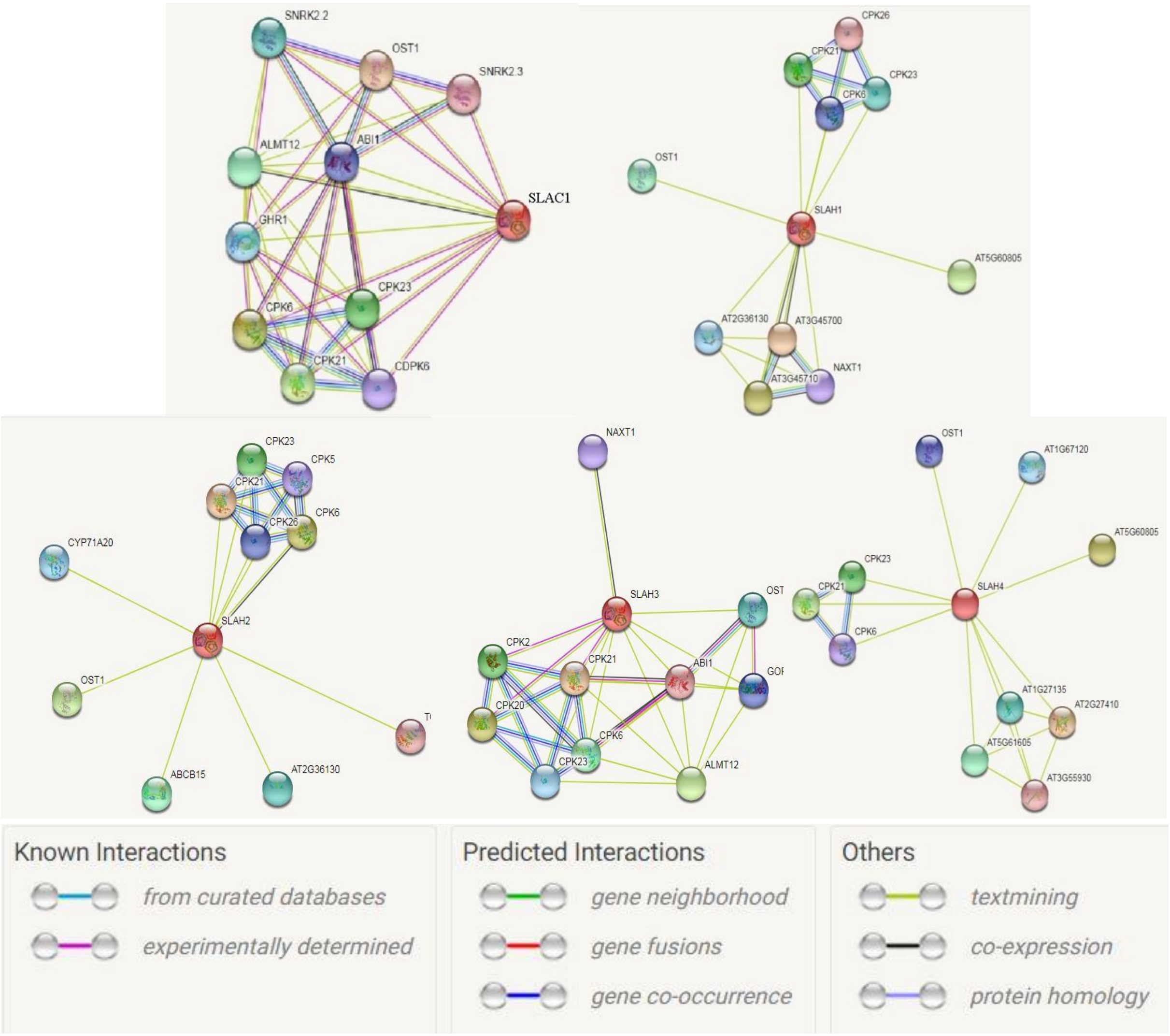

Figure S1. Prediction of SLAC/SLAH protein interaction in *Arabidopsis*.

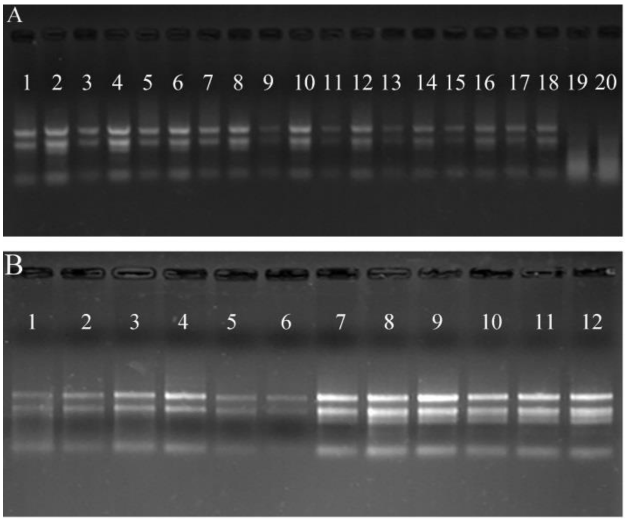

Figure S2. Electropherograms of RNA samples from high nitrogen level (HN，7.5 mM), low nitrogen level (LN，0.19 mM) and ABA treatment (100 μM).

A: nitrogen treatment; 1. 3h LN roots (R), 2. 3h LN leaves (L), 3. 3h HN R, 4. 3h HN L, 5. 12h LN R, 6. 12h LN L, 7. 12h HN R, 8. 12h HN L, 9. 24h LN R, 10. 24h LN L,11. 24h HN R, 12. 24h HN L, 13. 3d LN R, 14. 3d LN L, 15. 3d HN R, 16. 3d HN L, 17. 7d LN R, 18. 7d LN L, 19. 7d HN R, 20. 7d HN L;

B: ABA treatment; 1. 3h control roots (R), 2. 12h control R , 3. 24h control R, 4. 3h ABA R, 5. 12h ABA R, 6. 24h ABA R, 7. 3h control leaves (L), 8. 12h control L, 9. 24h control L, 10. 3h ABA L, 11. 12h ABA L, 12. 24h ABA L.

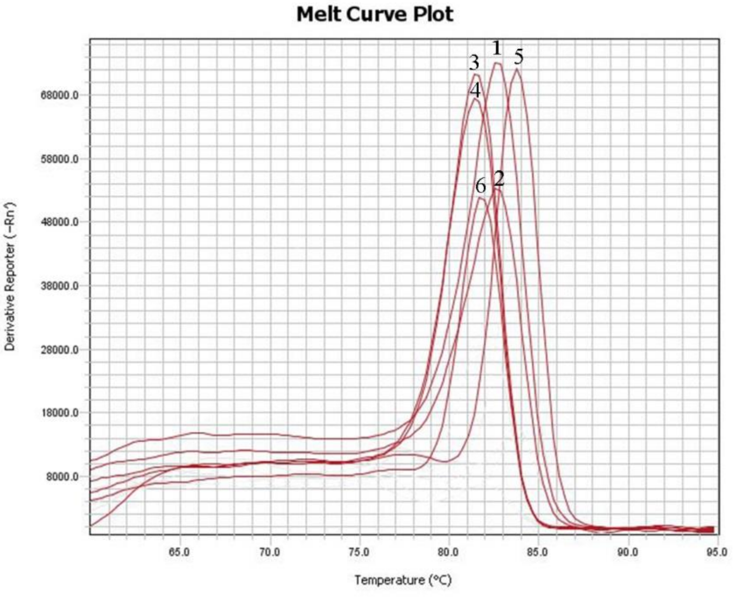

Figure S3. Melt curves of primers used for qRT-PCR

1. q*Bnactin7*-FP/RP; 2. q*BnSLAC1-1*-FP/RP; 3. q*BnSLAH1-1*-FP/RP; 4. q*BnSLAH3-2*-FP/RP; 5. q*BnSLAH3-3*-FP/RP; 4. q*BnSLAH3-4*-FP/RP;
